# Prediction of the Peak, Effect of Intervention, and Total Infected by COVID-19 in India

**DOI:** 10.1017/dmp.2020.321

**Published:** 2020-09-09

**Authors:** Parth Vipul Shah

**Affiliations:** Computer Science and Engineering, PES University, Bangalore, India

**Keywords:** COVID-19, India, infection rate, intervention, peak prediction, SEIR compartmental model

## Abstract

**Objectives:**

We study the effect of the coronavirus disease 2019 (COVID-19) in India and model the epidemic to guide those involved in formulating policy and building health-care capacity.

**Methods:**

This effect is studied using the Susceptible-Exposed-Infected-Recovered (SEIR) compartmental model. We estimate the infection rate using a least square method with Poisson noise and calculate the reproduction number.

**Results:**

The infection rate is estimated to be 0.270 and the reproduction number to be 2.70. The approximate peak of the epidemic will be August 9, 2020. A 25% drop in infection rate will delay the peak by 11 d for a 1-mo intervention period. The total infected individuals in India will be 9% of the total population.

**Conclusions:**

The predictions are sensitive to changes in the behavior of people and their practice of social distancing.

In this work, on the eve of the outbreak of the deadly coronavirus disease 2019 (COVID-19)^1^ in India, caused by severe acute respiratory syndrome coronavirus 2 (SARS-CoV-2),^2^ we predict the peak of the epidemic, effect of intervention, and total infected individuals. The objective of this study is to not only model the epidemic to study its effect but to also guide those involved in formulating policy and building health-care capacity. This disease has demonstrated person-to-person transmission,^3^ and as India is a densely populated country, it is necessary to understand the effects of this disease to mitigate its risk. January 30, 2020, marked the first identified case of COVID-19 in India.^4^ Meanwhile, COVID-19 has spread around the world. As of April 26, 2020, the United States of America has approximately 941 thousand confirmed cases, Spain has approximately 224 thousand confirmed cases, and Italy has approximately 195 thousand confirmed cases. India has approximately 27 thousand confirmed cases.^4^ The World Health Organization (WHO) declared the disease a pandemic on March 11, 2020.^5^ The total number of confirmed cases in India and the daily increase in cases is depicted below in Figures 1 and 2.

**FIGURE 1:**
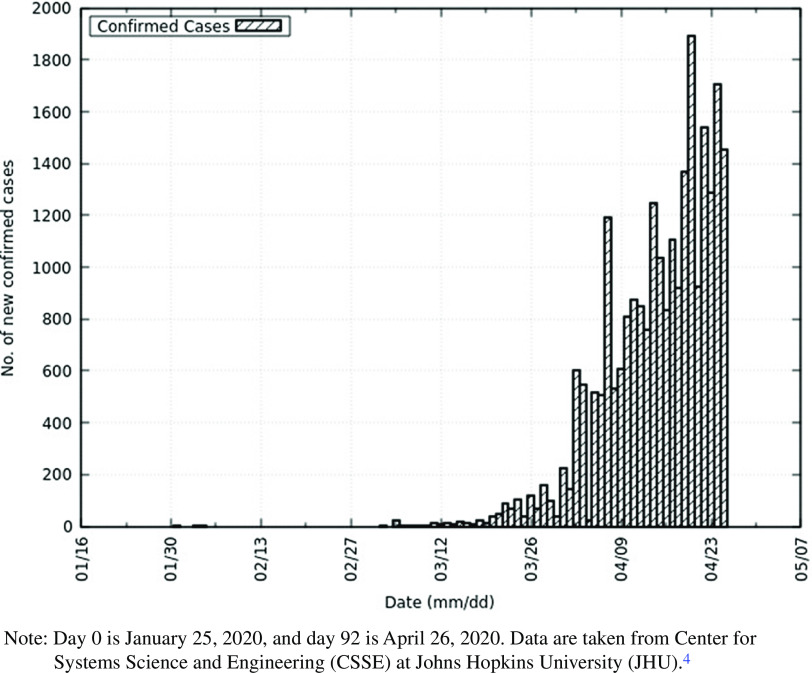
Daily Increase in Confirmed Cases of COVID-19 in India. Note: Day 0 is January 25, 2020, and day 92 is April 26, 2020. Data are taken from Center for Systems Science and Engineering (CSSE) at Johns Hopkins University (JHU).^4^

**FIGURE 2:**
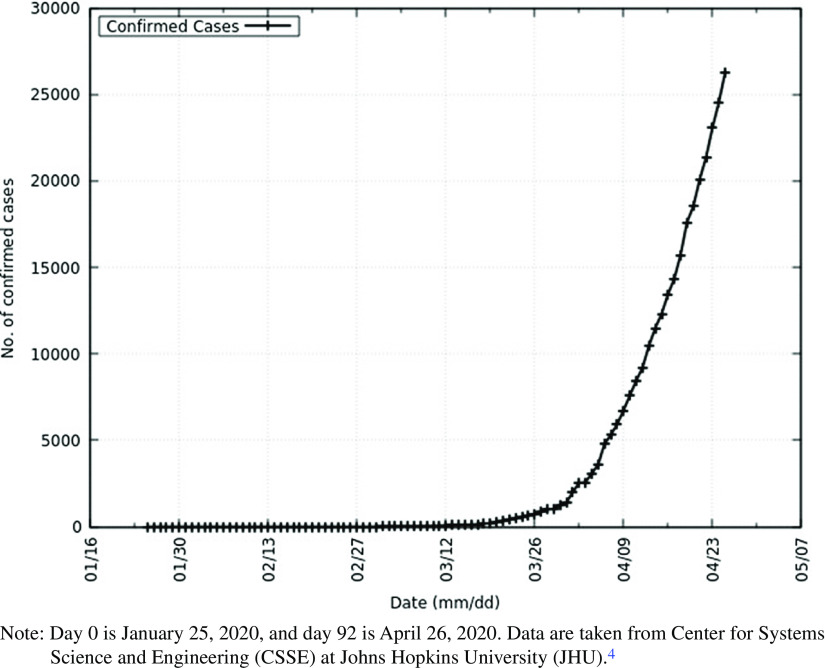
Cumulative Confirmed Cases of COVID-19 in India. Note: Day 0 is January 25, 2020, and day 92 is April 26, 2020. Data are taken from Center for Systems Science and Engineering (CSSE) at Johns Hopkins University (JHU).^4^

The growth of the number of confirmed COVID-19 cases can be approximated by an exponential function. The growth factor *r* at day *t* can be calculated as(1)
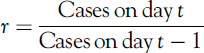



April 26, 2020, India is reporting an average growth rate of 1.1524 with a SD of 0.4882 (refer to Table 1). Stricter rules and measures of ensuring social distancing have been in place.^6^ India is fighting this disease by ensuring nation-wide lock-down and social distancing when the number of infected individuals is low. All nonessential workers across all industries have been given stay-at-home orders.

**TABLE 1:** Growth Factor, *r*

Date	No. of confirmed cases	*Growth factor, r*
April 22, 2020	1290	-
April 23, 2020	1707	1.323
April 24, 2020	1453	0.851
April 25, 2020	1753	1.206

## METHODS

### Model

The Susceptibel-Exposed-Infected-Recovered (SEIR) compartmental model has been used to model previous pandemics and diseases in the field of epidemiology. In Saito et al., it was used to model the 2009 influenza A (H1N1) pandemic in Japan.^7^ In Diaz et al., it was used to model the spread of Ebola in Western Africa.^8^ In addition to the SIR model,^9^ an additional compartment for the exposed population (“E”) is considered. The exposed population consists of individuals that are infected but not yet infectious and hence, introduces an incubation period for the virus in the model. Yang et al. in China and Kuniya in Japan use this model for the ongoing pandemic.^10,11^ The same methodology is adopted here. The assumption is that, once an individual contracts the virus and recovers, the individual is immune to this virus. The SEIR model is defined in Equation 2.(2)
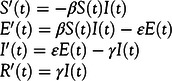



The susceptible, exposed, infected, and removed populations at time t is denoted by *S*(*t*)*, E*(*t*)*, I*(*t*)*, R*(*t*), respectively. The infection rate that is defined as the change in the number of infected individuals during the transition from the interval *t* − 1 to *t* is denoted by *β*. The onset rate, which is defined as the change in the number of individuals from infected to ill during the transition from the interval *t −* 1 to *t*, is denoted by *ε*. The removal rate, which is defined as the sum of recovery and death rates, is denoted by *γ*.^9,12^ The average incubation period and the average infectious period is 1*/ε* and 1*/γ*, respectively. We fix 1*/ε* to 5 and 1*/γ* to 10 based on recent publications that have studied the average incubation period and the average infectious period.^12,13^ The unit time is 1 d. The fraction of infected individuals that can be identified by diagnosis is denoted by *p*. We also fix *S* + *E* + *I* + *R* to 1 so that calculations are a proportion of the total population. The total population of India, N, is fixed at 1.33 billion or 1.3392 *×* 10^9^.^14^ The assumption that 1 individual among a total population of N individuals is identified as infected at *t* = 0 is adopted. Therefore, the total number of infected individuals who are identified at time t is given by the product of fraction of individuals that can be identified by diagnosis, infected individuals at time t and the total population (Equation 3).(3)
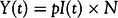



To obtain the initial conditions of the model, we assume that there are no exposed or removed populations at *t* = 0.(4)
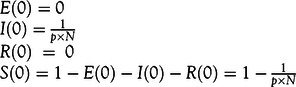
are the initial conditions. We fix the identification rate, *p* to 0.01 based on reports of low testing rates and high infection rates.^15^ The expected value of secondary cases produced by 1 infected individual, the reproduction number, *R*_0_ is calculated from Equation 5.^16^
(5)
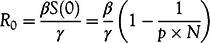



Using this model, we estimate the infection rate, *β*, in the next section.

### Estimation of the Infection Rate, *β*

Let *Y*(*t*), t = 0, 1, 2…92 be the daily confirmed cases of COVID-19 in India from January 25, 2020 (*t* = 0), to April 26, 2020 (*t* = 92). Using the least square approach with Poisson noise to estimate the infection rate, the following steps are adopted. With Poisson noise, Equation 3 is modified to(6)
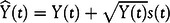

*s*(*t*)*, t* = 0, 1, 2*…*92 are random variables from a standard normal distribution. The following steps are adopted to estimate *β*.

1. For *β >* 0, calculate *Y* (*t*)*, t* = 0, 1, 2*…*92 using Equation 3.
2. Calculate 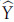 using Equation 6.
3. Calculate 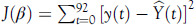.
4. Run step 1 to step 3 for 0 .2 *≤ β ≤* 0.4, and find *β* ^*^ such that *J* (*β* ^*^) = min_0.2*≤β≤*0.4_ *J*(*β*).
5. Repeat step 1 to step 4, 10000 times and obtain the distribution of *β* ^*^. Approximate the same by a normal distribution and obtain the 95% CI.

**FIGURE 3:**
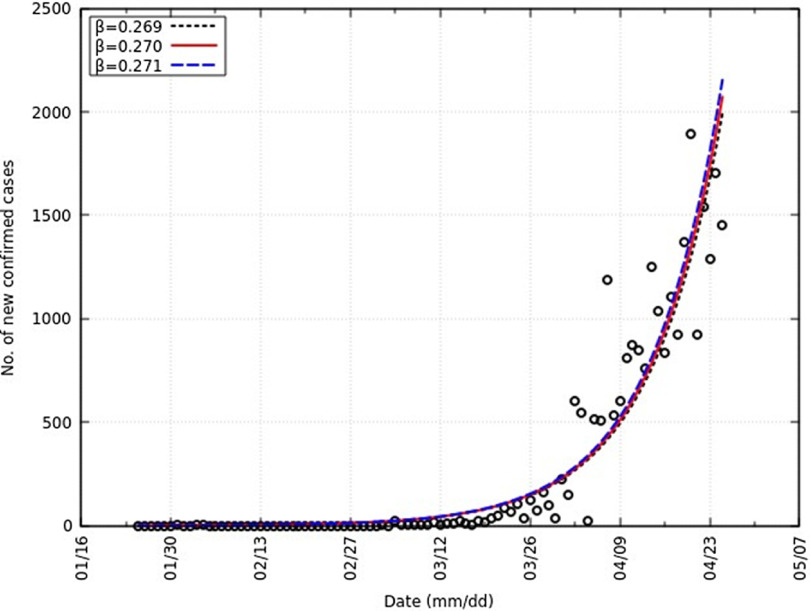
Comparison of Daily Confirmed Cases and *Y* in India From *t* = 0 to *t* = 92.

We obtain a value of *β* equal to 0.270 and the 95% CI as 0.269-0.271. Also, *R*_0_ is equal to 2.70 and the 95% CI as 2.69-2.71. Similar values of *R*_0_ have been reported (2.24-3.58).^17^ This is represented in Figure 3 with an overlap of the number of new confirmed cases. A summary of all parameters can be found in Table 2.

**TABLE 2:** Parameters

Parameter	Description	Value
*β*	Infection rate	0.270
*R*_0_	Reproduction number	2.70
*ε*	Onset rate	0.2
*γ*	Removal rate	0.1
*N*	Total population of India	1.3392 *×* 10^9^
*p*	Identification rate	0.01

## RESULTS

### Peak Prediction

The epidemic peak, *t* ^*^ is defined as the day t when the maximum value *Y* in a period of 550 d is achieved. As an expression, *Y* (*t* ^*^) = max_0*≤t≤*550_. As the epidemic peak and size are sensitive to the identification rate p, we report the following.

**FIGURE 4:**
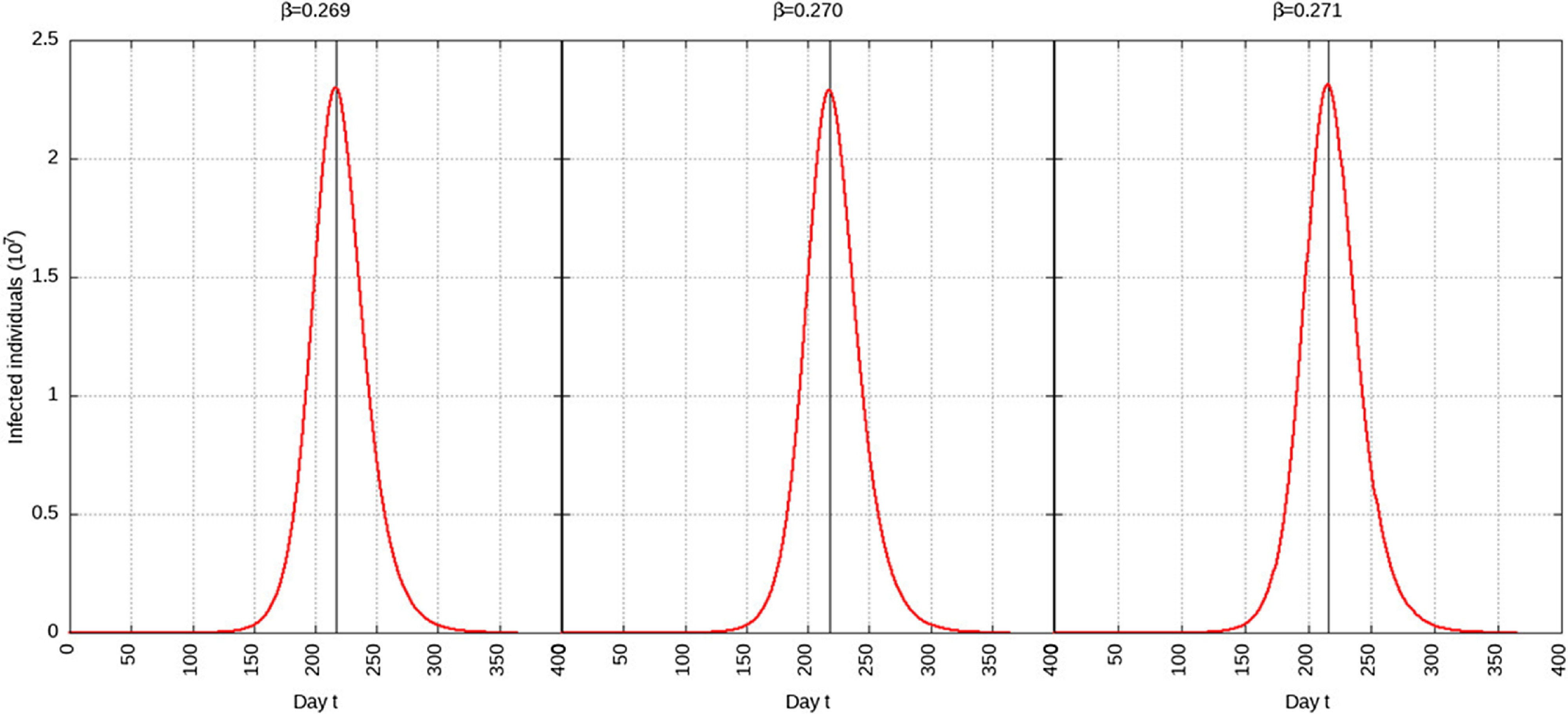
Infected Individuals for Time t, 0 *≤ t ≤* 550 for *p* = 0.1.

For *p* = 0.1, the estimated peak is *t* ^*^ = 218 with a 95% CI of 216-217. That is, starting from January 25, 2020 (*t* = 0), the estimated peak is September 4, 2020 (*t* = 218), and interval ranging from September 3, 2020, to September 5, 2020. This is represented in Figure 4.

**FIGURE 5:**
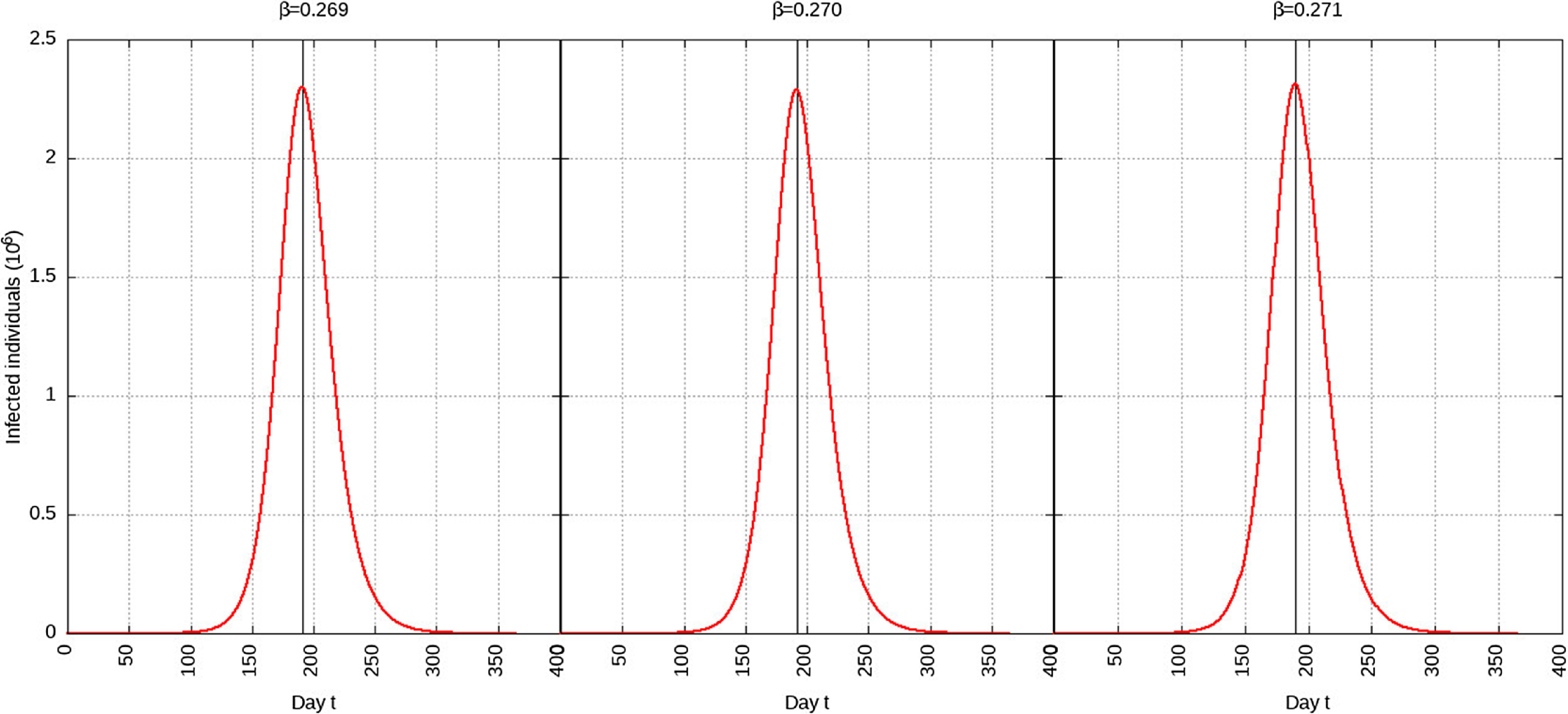
Infected Individuals for Time t, 0 *≤ t ≤* 550 for *p* = 0.01.

For *p* = 0.01, the estimated peak is *t* ^*^ = 192 with a 95% CI of 191-193. That is, starting with January 25, 2020 (*t* = 0), the estimated peak is August 9, 2020 (*t* = 192), and interval ranging from August 8, 2020, to August 10, 2020. This is represented in Figure 5.

### Effect of Intervention

India has announced a 21-d complete shutdown starting March 25, 2020.^18^ This was extended from April 15, 2020, to May 3, 2020.^19^ For our calculations, we assume that this shutdown will reduce the infection rate to 95%, 75%, or 50% of its original value of 0.270. For each reduction in infection rate, the effect of an intervention period of 30 d (1 mo) and 180 d (6 mo) is calculated. The government of India maintains that the third stage or community transmission has not begun.^20^ The current identification rate is low due to the low number of COVID-19 tests being conducted.^15^ Therefore, we set *p* to 0.01 as the current identification rate. This closely matches available data too.

#### Infection Rate Reduced to 95%

**FIGURE 6:**
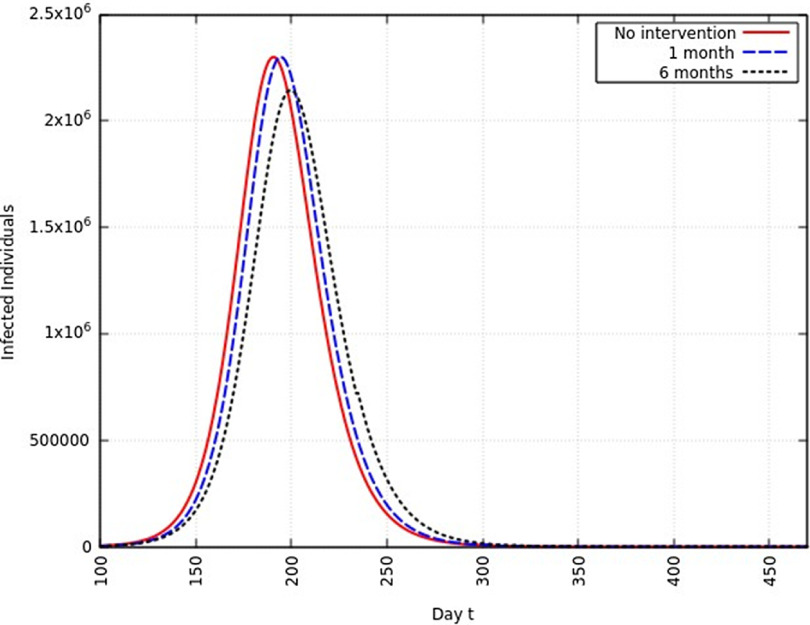
Variation in *Y* (*t*) for Tme t, 0 *≤ t ≤* 550 With no Intervention, 1 mo of Intervention, and 6 mo of Intervention With Assumption of *β* ^*j*^ = 0.95 *× β*. (*β* = 0.270).

When the infection rate is reduced to 95% and for 1 mo of intervention, the estimated peak is delayed by 3 d. That is, pushed from 192 d to 195 d since day 0 (January 25, 2020). When the infection rate is reduced to 95% and for 6 mo of intervention, the estimated peak is delayed by 8 d. That is, pushed from 192 d to 200 d since day 0 (January 25, 2020). These are represented in Figure 6.

#### Infection Rate Reduced to 75%

**FIGURE 7:**
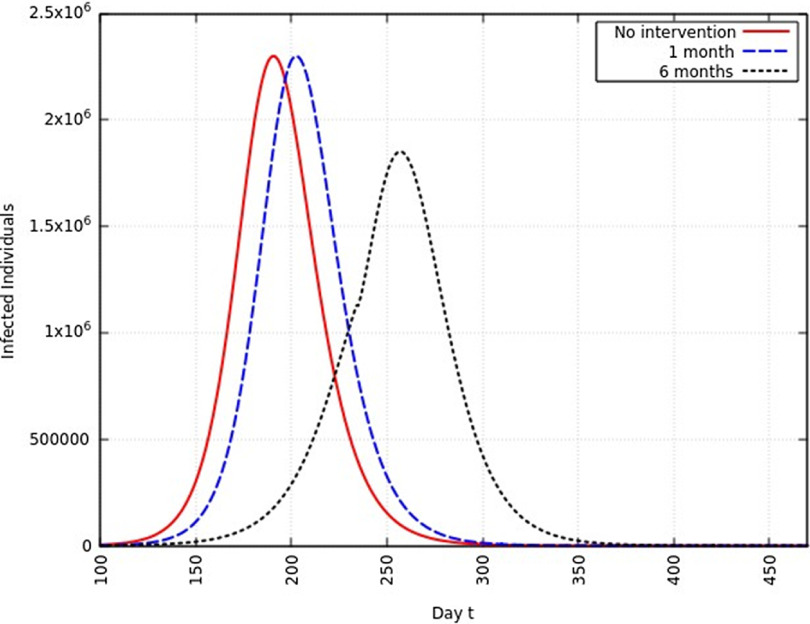
Variation in *Y* (*t*) for Time t, 0 *≤ t ≤* 550 With no Intervention, 1 mo of Intervention, and 6 mo of Intervention With Assumption of *β* ^*j*^ = 0.75 *× β*. (*β* = 0.270).

When the infection rate is reduced to 75% and for 1 mo of intervention, the estimated peak is delayed by 11 d. That is, pushed from 192 d to 203 d since day 0 (January 25, 2020). When the infection rate is reduced to 75% and for 6 mo of intervention, the estimated peak is delayed by 65 d. That is, pushed from 192 d to 257 d since day 0 (January 25, 2020). This is represented in Figure 7.

#### Infection Rate Reduced to 50%

**FIGURE 8:**
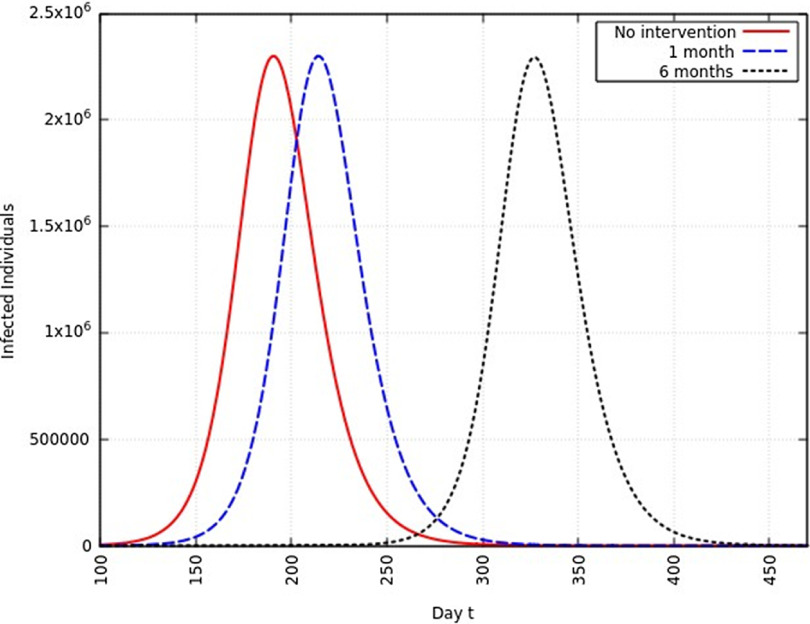
Variation in *Y* (*t*) for Time t, 0 *≤ t ≤* 550 With no Intervention, 1 mo of Intervention and 6 mo of Intervention With Assumption of *β* ^*j*^ = 0.50 *× β*. (*β* = 0.270).

When the infection rate is reduced to 50% and for 1 mo of intervention, the estimated peak is delayed by 22 d. That is, pushed from 192 d to 214 d since day 0 (January 25, 2020). When the infection rate is reduced to 50% and for 6 mo of intervention, the estimated peak is delayed by 135 d. That is, pushed from 192 d to 327 d since day 0 (January 25, 2020). This is represented in Figure 8.

**FIGURE 9:**
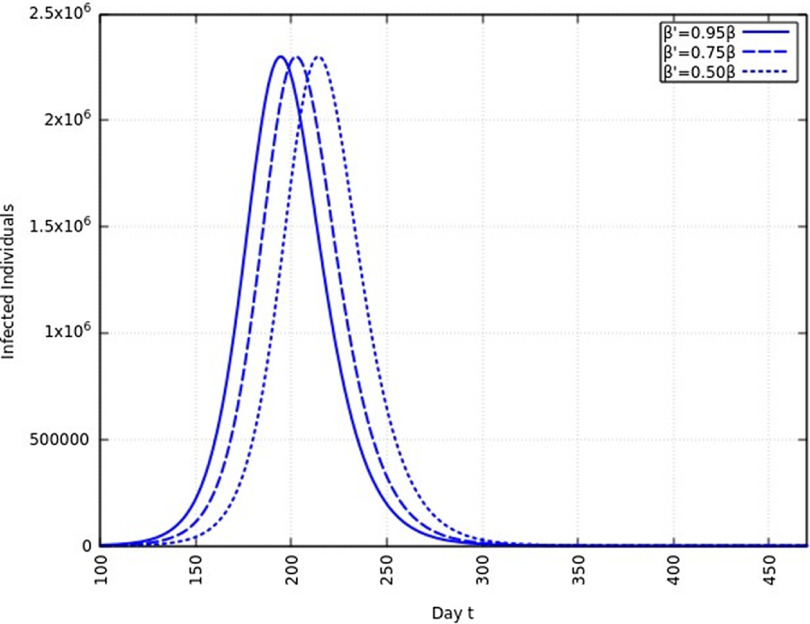
Variation in *Y* (*t*) for Time t, 0 *≤ t ≤* 550 With 1 mo of Intervention and With Assumption of *β* ^*j*^ = 0.50 *× β, β* ^*j*^ = 0.75 *× β, β* ^*j*^ = 0.95 *× β*. (*β* = 0.270).

**FIGURE 10:**
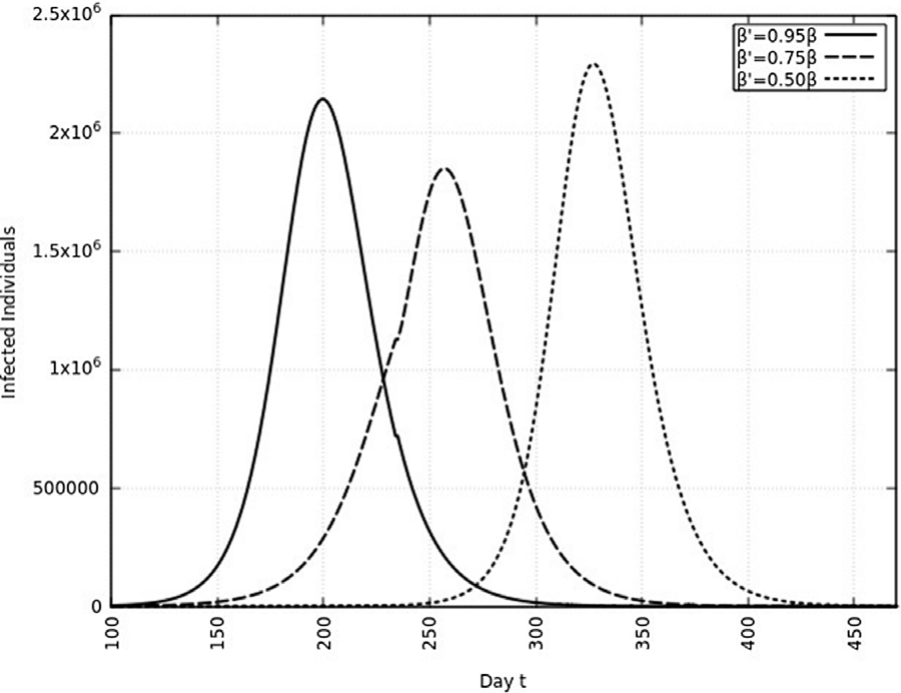
Variation in *Y* (*t*) for Time t, 0 *≤ t ≤* 550 With 6 mo of Intervention and With Assumption of *β* ^*j*^ = 0.50 *× β, β* ^*j*^ = 0.75 *× β, β* ^*j*^ = 0.95 *× β*. (*β* = 0.270).

A summary of all the delays in the estimated peaks can be found in Table 3. Additionally, Figures 9 and 10 depict the change in total infected individuals for 1 mo and 6 mo of intervention, respectively.

**TABLE 3:** Summary of Change in Estimated Peak From August 9, 2020^a^

*β* Drop	1-mo Delay	Date	6-mo Delay	Date
95%	3	August 12	8	August 17
75%	11	August 20	65	October 13
50%	22	August 31	135	December 22

a Units are days. The 1-mo and 6-mo delays are the delays in the estimated peaks after respective intervention periods. The dates indicated are the revised dates of the estimated peaks in 2020.

### Total Infected Individuals

Upon integrating *Y* (*t*) from Equation 3 over time *t*, we obtain an approximate of the total number of infected individuals over 550 d. With no intervention, the approximate total number of infected individuals will be 1.2261 *×* 10^8^ which is 9% of the total population of India. With 1 mo of intervention and infection rate reduced to 75%, the approximate total number of infected individuals will be the same. But with 6 mo of intervention and infection rate reduced to 75%, the approximate total number of infected individuals will be 1.2143 *×* 10^8^ compared with 1.2261 *×* 10^8^ with no intervention. This means approximately 1.1800 *×* 10^6^ individuals will not be infected. A higher reduction in infection rate does not significantly change the approximate total number of infected individuals. It only pushes the approximate epidemic peak to a later date.

**FIGURE 11:**
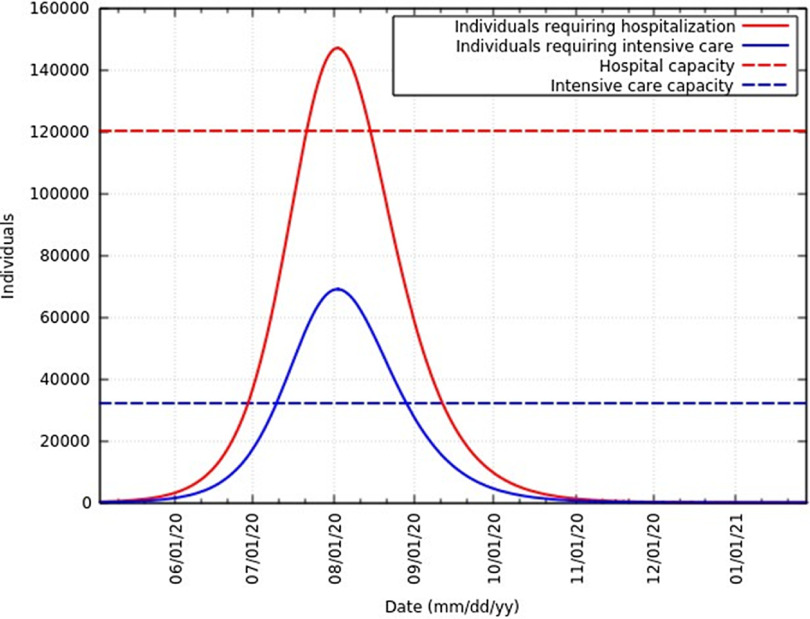
Infected Individuals Requiring Hospitalization or Intensive Care and Health-Care Capacities in India.

Of the total infected individuals, approximately 6.39% require hospitalization and 3% require intensive care.^21^ Figure 11 represents the total infected individuals with a need for hospitalization and intensive care. These rates are in agreement with rates reported in the United States of America,^22^ Northern Italy,^23^ and globally. As of June 12, 2020, Ministry of Health and Family Welfare, Government of India sources say 120,104 hospital beds and 32,362 intensive care beds are dedicated to the fight against this epidemic.^24^ Figure 11 also represents the current hospital and intensive care capacity. At the peak, infected individuals will exceed hospital capacity by approximately 27,000 and intensive care capacity by approximately 37,000. To avoid exceeding hospital and intensive care capacity at the peak, the infection rate needs to be 0.236 (reduced to 87% of the original value) and 0.181 (reduced to 67% of the original value), respectively. However, hospital and intensive care capacity is constantly being increased. A summary of these data at the peak is presented in Table 4.

**TABLE 4:** Total Number of Infected Individuals (II), Individuals Requiring Hospitalization (RH), and Individuals Requiring Intensive Care (RIC) With Different Reductions in *β* ^a^

*β* Drop	Parameter	1-mo Delay	6-mo Delay
95%	II	2.30 *×* 10^6^	2.14 *×* 10^6^
	RH	146971	137038
	RIC	69000	64337
75%	II	2.30 *×* 10^6^	1.85 *×* 10^6^
	RH	146982	118345
	RIC	69005	55561
50%	II	2.30 *×* 10^6^	2.29 *×* 10^6^
	RH	146998	146621
	RIC	69013	68836

a Numbers represented are at the peak. The total infected individuals without any intervention is 2.30 *×* 10^6^.

## DISCUSSION

From this work, by applying the SEIR compartmentalization model, it is clear that the COVID-19 epidemic peak can easily reach August 2020. This prediction is sensitive to changes in the behavior of not only the virus but of people and their practice of social distancing. India is still in the process of gearing up its health-care system, and it currently may be ill-equipped to deal with a large number of cases. Delaying the peak will ensure adequate medical equipment and personnel for all her citizens. It must be noted that, while a large reduction in infection rate or, *β*, is effective in delaying the peak, without continual intervention and efforts to reduce *β*, the total number of infected individuals does not change significantly. After a period of intervention, *β* is assumed to go back to the originally calculated value. Therefore, intervention over a relatively long period is needed to effectively reduce the final epidemic size. In light of this, the WHO has issued multiple statements reiterating the importance of practicing social distancing. Additionally, proper hand hygiene, effective contact tracing and the use of face coverings are recommended.^5^

This model is without vital dynamics that models births and natural deaths in a population in addition to the epidemic. An assumption made in this model is that the onset rate, removal rate, and the identification rate are all constant during the epidemic. As stated in the Methods section, it is assumed that complete immunity is conferred by a single infection.

Others have attempted to model the epidemic. In Mandal et al., a modified SEIR model is used to analyze the epidemic dynamics in 4 metropolitan districts based on population connectivity.^25^ Optimistic and pessimistic estimates of the reproduction number, *R*_0_, are fixed to 1.5 and 4, respectively. However, this model fails to take into account the rural population of India and calculates dynamics using estimated reproduction numbers.

The burden of COVID-19 may disproportionately fall on disadvantaged or vulnerable individuals or groups. These include but are not limited to the elderly, people with disabilities, communities living in remote locations with little access to health care, and patients with chronic diseases or comorbidities. These groups have been identified by the Ministry of Health and Family Welfare, Government of India.^26^ However, this model does not stratify the population based on age, access to health care, chronic diseases, or comorbidities. It also assumes that the entire population is initially equally susceptible. COVID-19 may be transmitted by asymptomatic carriers.^27^ This would lead to a higher *β*. This model does not directly account for asymptomatic carriers, but the value of the identification rate, *p*, can be modified as asymptomatic carriers are a fraction of the infected population. The model and the estimation of *β* is sensitive to the parameters listed in Table 2, and uncertainties surround its true values as COVID-19 is a new disease.
